# Preparation of Quasi-Three-Dimensional Porous Ag and Ag-NiO Nanofibrous Mats for SERS Application

**DOI:** 10.3390/s18092862

**Published:** 2018-08-30

**Authors:** Huixiang Wu, Xiangcheng Sun, Changjun Hou, Jingzhou Hou, Yu Lei

**Affiliations:** 1 Key Laboratory for Biorheological Science and Technology of Ministry of Education, State and Local Joint Engineering Laboratory for Vascular Implants, Bioengineering College of Chongqing University, Chongqing 400044, China; huixiang.wu@uconn.edu; 2 Department of Chemical and Biomolecular Engineering, University of Connecticut, Storrs, CT 06269, USA; 3 Department of Chemistry and Chemical Biology, Cornell University, Ithaca, NY 14853, USA; xs266@cornell.edu; 4 Liquor Making Biology Technology and Application of Key Laboratory of Sichuan Province, College of Bioengineering, Sichuan University of Science and Engineering, Zigong 643000, China; 20161701001@cqu.edu.cn; 5 Department of Biomedical Engineering, University of Connecticut, Storrs, CT 06269, USA

**Keywords:** electrospinning, silver, nanofiber, surface enhanced Raman scattering, melamine, methyl parathion

## Abstract

In this study, two new quasi-three-dimensional Surface Enhanced Raman Scattering (SERS) substrates, namely porous Ag and Ag-NiO nanofibrous mats, were prepared using a simple, electrospinning-calcination, two-step synthetic process. AgNO_3_/polyvinyl pyrrolidone (PVP) and AgNO_3_/Ni(NO_3_)_2_/PVP composites serving as precursors were electrospun to form corresponding precursory nanofibers. Porous Ag and Ag-NiO nanofibers were successfully obtained after a 3-h calcination at 500 °C under air atmosphere, and analyzed using various material characterization techniques. Synthesized, quasi-three-dimensional porous Ag and Ag-NiO nanofibrous mats were applied as SERS substrates, to measure the model compound Rhodamine 6G (R6G), and investigate the corresponding signal enhancement. Furthermore, porous Ag and Ag-NiO nanofibrous mats were employed as SERS substrates for melamine and methyl parathion respectively. Sensitive detection of melamine and methyl parathion was achieved, indicating their feasibility as an active SERS sensing platform, and potential for food safety and environmental monitoring. All the results suggest that the electrospinning-calcination, two-step method offers a new, low cost, high performance solution in the preparation of SERS substrates.

## 1. Introduction

Raman spectroscopy is an accurate and attractive molecule identification and monitoring method. It probes the chemical contents through molecular vibration, providing a unique, specific chemical or vibration “finger-print” for molecules [1,2,3,4]. However, its relatively low sensitivity poses challenges in trace chemical detection. Therefore, since its discovery in the 1970s surface enhanced Raman scattering (SERS) has become a hot research topic due to its enhanced signal amplification [1,5,6,7,8]. SERS is a phenomenon originating from a giant enhancement of the electromagnetic field surrounding noble nanostructured materials (such as Ag or Au). Generally, two main mechanisms, namely electromagnetic enhancement and chemical enhancement, are used as reasonable explanations for the observed enhanced Raman signals. On the one hand, excitation of localized, surface plasmon resonance enables light amplification, resulting in electromagnetic enhancement. On the other hand, excitation wavelength, resonating with metal-molecule charge transfer, offers a significant chemical enhancement.

The uniqueness of SERS technology can be ascribed to its ability to obtain structural information through molecule vibrations within a broad wavelength range. Therefore the discovery of SERS has resulted in a new sensor research field. SERS based sensors demonstrated several benefits, especially in ultra-sensitivity (enhanced signals) and excellent selectivity (fingerprint spectrum) in real applications, compared with traditional analytical methods [9,10,11,12]. SERS has already been applied in analytes detection with single molecule sensitivity [13]. As one of today’s most sensitive analytical techniques, it was applied not only in fundamental research, but also for analytical applications in biomedical and environmental areas [14].

For SERS sensors, fabrication of SERS substrates is of paramount importance in effective, sensitive and reproducible detection of targets. Consequently, the preparation of highly efficient SERS substrates has drawn considerable attention [15]. Aggregated colloidal nanoparticles with various shapes and sizes, prepared by a wet chemistry method or roughened electrode surfaces, were extensively reported and utilized as SERS substrates for target analysis [16,17,18]. However, this kind of SERS substrate generally lacks control of the surface morphology. In order to fabricate large-scale, reproducible, and highly controlled SERS substrates, a variety of techniques have been applied, including vacuum evaporation [19], physical vapor deposition [20], electron beam lithography [21], nanosphere lithography [22], focused ion beam patterning [23], etc. Well-ordered, noble metal nanostructure-based SERS substrates, with better stability, reproducibility and sensitivity could be achieved. However, the high cost, long preparation time, and bulky instruments required, greatly hinder their wide application. Therefore, there is a need to develop a simple and economical way to prepare SERS substrates within a highly-controlled structure.

Electrospinning provides a novel, simple and template-free strategy to prepare quasi-3D polymer nanofibrous membranes [24,25,26]. Nanofibers with a homogenous diameter can be generated because electrostatic forces continuously stretch the viscous precursor solution at the electrified jet [27,28,29]. Currently, various quasi-3D polymer nanofibrous matrixes are prepared with electrospinning, followed by decoration with noble metal nanostructured materials to form SERS substrates [26,30,31,32,33]. However, inhomogeneous distribution of the noble metal nanostructured materials on the nanofibrous matrixes would influence the efficiency of target analysis. Yu et al. uniformly mixed poly(vinyl alcohol) (PVA) with a certain amount of Ag nanoparticles or Au nanorods to form an homogeneous gel, subsequently endowing the preparation of large-scale, flexible, free-standing SERS substrates through electrospinning [25,34]. SERS detections were consequently realized by accessing Ag/Au nanomaterials through target molecules penetrating into the PVA. Nevertheless, detection of targets was potentially affected by the presence of PVA, leading to a long diffusion distance, and blocking between target molecules and nanomaterials substrates, resulting in low Raman signal enhancement.

**Figure 1:**
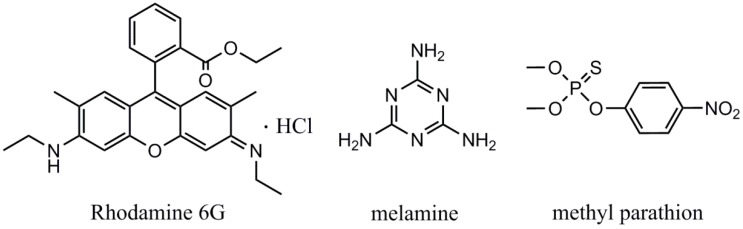
Chemical structures of Rhodamine 6G, melamine and methyl parathion.

Previously, we prepared quasi-3D porous Ag and Ag-NiO nanofibrous mats simply by an electrospinning-calcination, two-step synthesis route, in which AgNO_3_ and AgNO_3_/Ni(NO_3_)_2_ served as precursors [35]. The nanofibrous mats were then employed for the detection of glucose using an electrochemical method. It was found that formation of NiO could greatly maintain the fibrous structure after calcination. The synthesized porous Ag displayed a rough, large, specific surface area, while Ag-NiO nanofibrous mat demonstrated a uniform structure and ahomogenous distribution of Ag nanophase. Therefore, these two materials hold great potential in serving as novel SERS substrates for trace chemical detection. In this study, we prepared porous Ag and Ag-NiO nanofibrous mats, which were then used as SERS substrates. Enhancement factor (EF) of porous Ag and Ag-NiO nanofibers mats were evaluated using Rhodamine 6G (shown in Figure 1). Later, two model compounds, methyl parathion (an organophosphorus pesticide) and melamine (an illegal food additive) (Figure 1), were used to validate the applicability of the as-fabricated SERS sensing materials in food safety and environmental monitoring. These results suggest that the electrospinning-calcination, two-step method offers a new, low cost, high performance route in the preparation of SERS substrates.

## 2. Experimental Section

### 2.1. Reagents and Chemicals

Nickel nitrate hexahydrate (Ni(NO_3_)_2_·6H_2_O) and silver nitrate (AgNO_3_) were bought from Acros Organics. Rodamine 6G (R6G), methyl parathion (C_8_H_10_NO_5_PS), melamine (C_3_H_6_N_6_) and poly(vinyl pyrrolidone) (PVP, MW ¼ 1,300,000) were acquired from Sigma-Aldrich. All chemicals were of analytical grade and used without any pretreatment. Ultrapure water (18.2 MΩ∙cm resistivity) was employed to prepare aqueous solutions.

### 2.2. Instruments and Apparatus

Scanning electron microscopic (SEM) images were recorded using FEI Tecnai G2 Spirit BioTWIN and FEI Nova NanoSEM 450. X-ray Diffraction (XRD) pattern was recorded by a Rigaku Ultima IV diffractometer. A portable Raman spectrometer (QE Pro, Ocean Optics) was used to collect the Raman spectra coupled with a 785 nm, 499 mW laser. For each measurement, the Raman spectrum was obtained with an integration time of 5 s.

### 2.3. Preparation of Quasi-3D Porous Ag and Ag-NiO Nanofibers Mats

**Figure 2:**
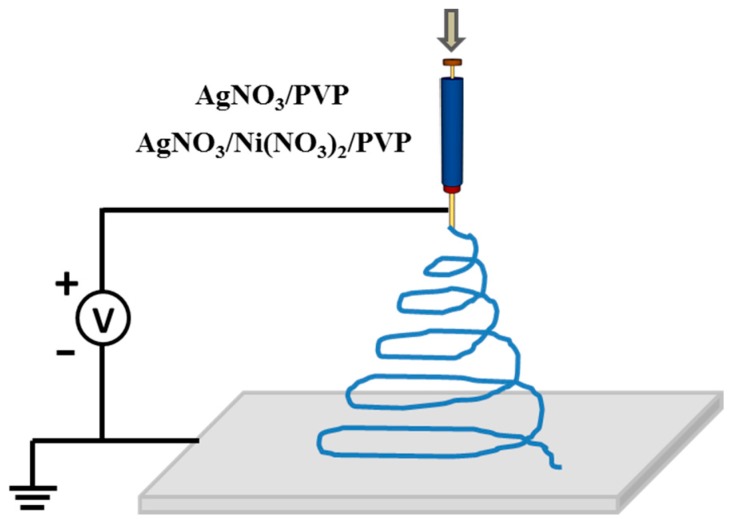
Illustration of electrospinning process.

Quasi-3D porous Ag and Ag-NiO nanofibrous mats were synthesized following the procedure in our previous report, with a minor revision [35]. In a typical process for obtaining Ag-NiO nanofibrous mats, 0.2 g AgNO_3_, 0.2 g Ni(NO_3_)_2_·6H_2_O and 0.8 g PVP were dissolved in 4 mL dimethyl formamide (DMF). The mixture was then stirred for 4 h to form an homogenous solution. Figure 2 shows the electrospinning of nanofibers. The AgNO_3_/Ni(NO_3_)_2_/PVP nanofibers were prepared using the electrospinning setup, in which a 23-gauge needle and flow rate of 0.3 mL/h was employed, with an applied voltage of 20 kV. The collection distance between the needle tip and aluminum foil (serving as nanofibers collector) was 15 cm. In order to acquire the Ag-NiO nanofiber mats, as-synthesized AgNO_3_/Ni(NO_3_)_2_/PVP nanofibers were then thermal-treated at 500 °C for 3 h under air atmosphere. Preparation of porous Ag followed a similar procedure except for the absence of nickel salt (Ni(NO_3_)_2_·6H_2_O).

### 2.4. Sample Preparation and Measurement Procedure

A methyl parathion ethanolic solution (0.01 M), R6G (0.01 M) and melamine (0.01 M) aqueous solution were prepared and used as stock solutions. Methyl parathion, R6G and melamine solutions with various concentrations were obtained by diluting corresponding stock solutions. Target molecule solutions (2 μL) with certain concentrations were directly dropped onto the surface of porous Ag. After drying of the target solutions the SERS spectra were recorded. To fabricate Ag-NiO nanofiber mats-based substrate, 0.2 mg of Ag-NiO nanofibers was dispersed into 2 mL water and treated with an ultrasonic bath for 30 s. Then, 5 μL of suspension was dropped onto a silicon wafer and left to dry. Two microliters of target solution with various concentrations were dropped onto the Ag-NiO nanofibers SERS substrate, and after being dried on the surface the SERS spectra was recorded.

## 3. Results and Discussions

### 3.1. Nanofber Characterization

**Figure 3:**
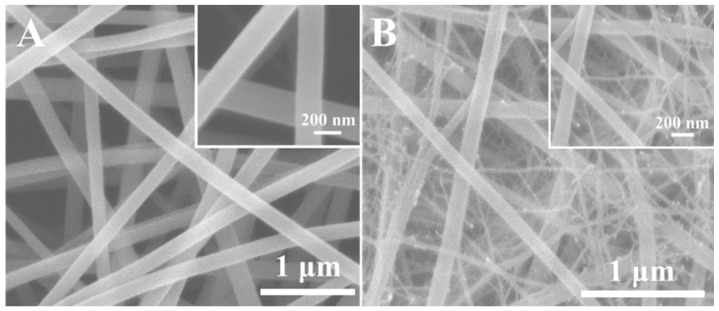
Representative SEM images of (**A**) AgNO_3_/PVP nanofibers and (**B**) AgNO_3_/Ni(NO_3_)_2_/PVP nanofibers, respectively. The insets show corresponding SEM images with a higher magnification.

To study the morphology of the electrospun nanofibers before and after calcination, SEM characterization was first conducted. Figure 3A shows the typical morphology of the electrospun AgNO_3_/PVP nanofibers. The AgNO_3_/PVP nanofibers possess smooth surface and good uniformity. The inset of Figure 3A indicates that the average diameter of AgNO_3_/PVP nanofibers was about 200 nm. Figure 3B indicates that similar morphology was obtained for the as-prepared AgNO_3_/Ni(NO_3_)_2_/PVP nanofibers with a smaller average diameter (ca. 150 nm). However, there are some very tiny nanowires intertwined with large nanofibers. These observations can mainly be attributed to the difference in electrical conductivity and viscosity between AgNO_3_/PVP and AgNO_3_/Ni(NO_3_)_2_/PVP precursors. These results indicate the successful synthesis of AgNO_3_/PVP nanofibers and AgNO_3_/Ni(NO_3_)_2_/PVP nanofibers using electrospinning method.

After 3 h of thermal treatment of the precursory nanofibers at 500 °C in air, the polymer completely decomposed and disappeared. The AgNO_3_ and Ni(NO_3_)_2_ were degraded to yield metal Ag and NiO, respectively, following the reactions below [35,36]:

(1) $$2AgNO_{3}\overset{\Delta }{\to }2Ag+2NO_{2}↑+O_{2}↑$$

(2) $$2Ni{\left(NO_{3}\right)}_{2}\overset{\Delta }{\to }2NiO+4NO_{2}↑+O_{2}↑$$

**Figure 4:**
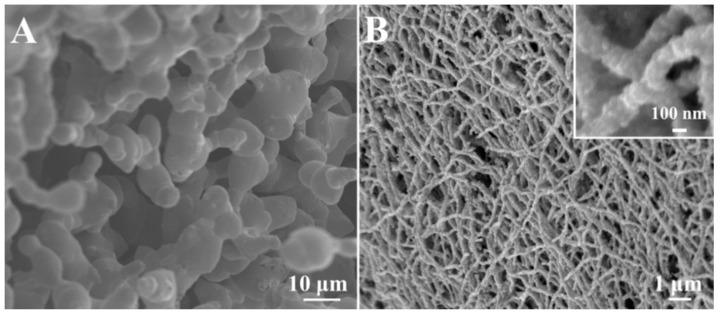
Typical SEM images of (**A**) porous Ag and (**B**) Ag-NiO nanofibers, respectively. The insets show the SEM image with a higher magnification.

Quasi-3D porous Ag mat was obtained after calcination of AgNO_3_/PVP nanofibers. However, due to the high temperature applied during calcination, some Ag merged together to form 3D porous structure with a rough rather than a fibrous surface, shown in Figure 4A. By contrast, NiO still maintained the nanofiber structure at 500 °C, leading to well-defined Ag-NiO nanofibers (Figure 4B). It can be observed that as-synthesized Ag-NiO nanofibers displayed a rough surface, which was attributed to the decomposition of PVP, metal (Ag) crystallization and metal oxide (NiO) formation. High magnification SEM image further confirmed the formation of Ag-NiO nanofibers with rough surfaces (inset of Figure 4B). Quasi-3D porous Ag porous network and Ag-NiO nanofiber mats with rough surface structures potentially offer a large surface area and a number of hot spots, which render them active and efficient SERS substrates for sensing applications.

**Figure 5:**
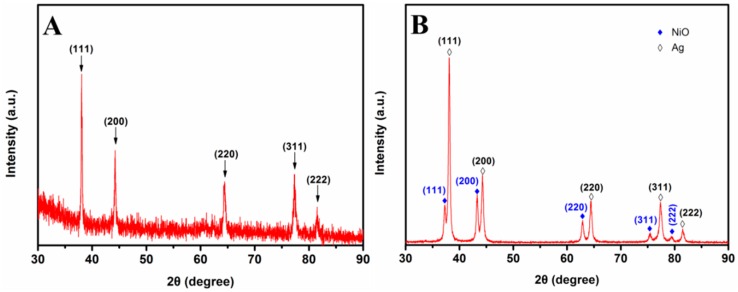
XRD patterns of (**A**) porous Ag and (**B**) Ag-NiO nanofibers, respectively.

To study the chemical composition and crystallinity of porous Ag network and Ag-NiO nanofibers, XRD study was conducted. Figure 5A shows the XRD spectrum collected from 30° to 90° of porous Ag mat. Five sharp and strong diffraction peaks at 2θ of 38.06°, 44.26°, 64.44°, 77.28° and 81.48° were observed, corresponding to (111), (200), (220), (311) and (222) crystal planes of Ag, respectively. This result indicates the formation of cubic crystalline Ag [35]. The XRD pattern of Ag-NiO composite is shown in Figure 5B. Beside the diffraction peaks of Ag, peaks at 2θ of 38.28°, 43.30°, 62.90°, 75.48° and 79.42° appeared, which correspond with (111), (200), (220), (311) and (222) crystal planes of NiO. The XRD results demonstrate the formation of Ag and NiO [35]. These observations indicate the successful synthesis of porous Ag network and Ag-NiO composite nanofibers.

### 3.2. SERS Performance of Porous Ag and Ag-NiO Nanofibers

**Figure 6:**
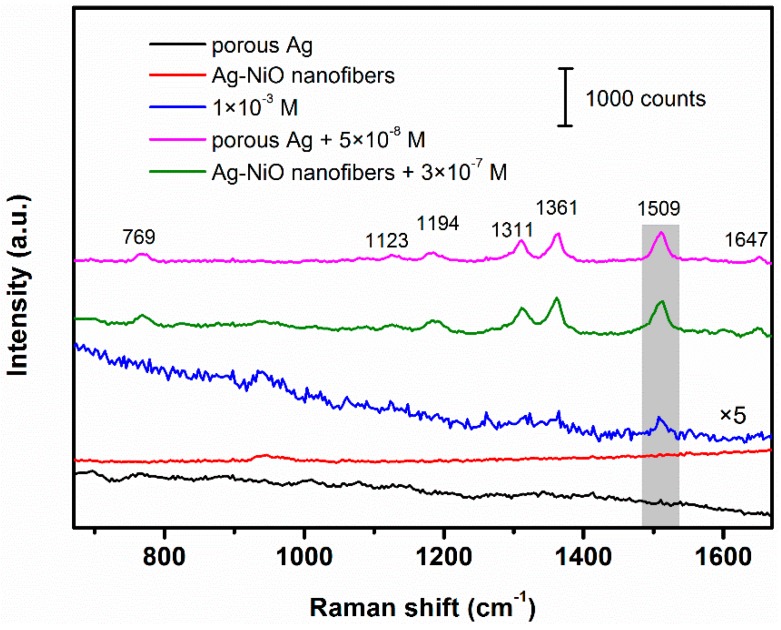
Raman spectra of porous Ag, Ag-NiO nanofibers and R6G at 1 × 10^−^^3^ M and SERS spectra of R6G at 5 × 10^−^^8^ M and 3 × 10^−^^7^ M recorded on porous Ag and Ag-NiO nanofibers, respectively.

To study the SERS activity of as-synthesized porous Ag and Ag-NiO nanofibrous mats, R6G was used as a model Raman dye. Figure 6 shows the Raman spectrum of R6G (5 times) with concentration of 1 × 10^−^^3^ M, SERS spectra of R6G with concentration of 5 × 10^−^^8^ M and 3 × 10^−^^7^ M recorded on the porous Ag and Ag-NiO nanofibers mats, respectively (Raman shift in the range from 670 cm^−1^ to 1670 cm^−1^ was collected). There was no obvious Raman scattering for both porous Ag and Ag-NiO nanofibers without casting R6G, indicating negligible background interferences from as-fabricated SERS substrates. Raman spectrum of high concentration R6G shows three relatively weak peaks at 1311 cm^−1^, 1361 cm^−1^ and 1509 cm^−1^. By contrast, SERS spectrum of R6G with much lower concentration was collected on the porous Ag. Besides three aforementioned peaks, four other distinct peaks appeared at 769 cm^−1^, 1123 cm^−1^, 1194 cm^−1^ and 1647 cm^−1^, accompanied with a significantly enhanced Raman signal. All these molecule vibration assignments were listed in Table 1 [37,38,39,40]. Similar Raman signal enhancement on Ag-NiO nanofibrous mats was observed, except for the degree of enhancement. The enhancement factor (EF) of porous Ag and Ag-NiO nanofibrous mat was determined using the following expression [41,42]:

(3) $$\mathrm{EF}=\frac{\left(I_{SERS}/C_{SERS}\right)}{\left(I_{NRS}/C_{NRS}\right)}$$

where $I_{SERS}$ and $I_{NRS}$ are the integrated SERS and normal Raman scattering (NRS) intensities of R6G at the same Raman band, respectively. $C_{SERS}$ and $C_{NRS}$ are the concentrations of probed molecules in the SERS and NRS measurements, respectively. In this study, Raman intensities with baseline correction of R6G at 1509 cm^−1^ were extracted to serve as $I_{SERS}$ and $I_{NRS}$. Values of $I_{SERS}$, $I_{NRS}$, $C_{SERS}$ and $C_{NRS}$ for both of porous Ag and Ag-NiO nanofibers were summarized in Table 2. The as-prepared porous Ag and Ag-NiO nanofibers show EF of 1.59 × 10^5^ and 2.89 × 10^4^ for R6G. A relative lower EF obtained for Ag-NiO nanofibers can be attributed to the distribution of NiO phase on the surface of Ag-NiO nanofibers [35].

### 3.3. SERS Detection for Melamine and Methyl Parathion

**Figure 7:**
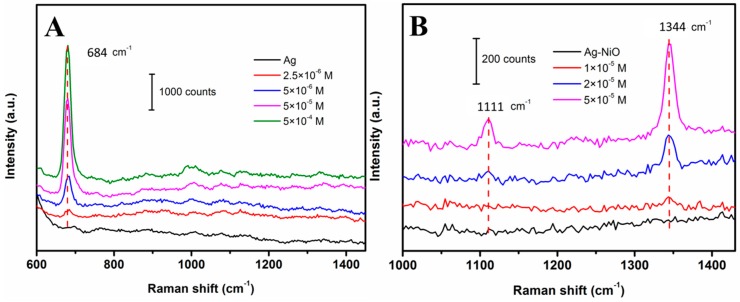
SERS spectra with various concentrations of (**A**) melamine recorded on porous Ag and (**B**) methyl parathion recorded on Ag-NiO nanofibers, respectively.

To further demonstrate the applicability of porous Ag and Ag-NiO nanofibers mat in SERS sensing, porous Ag was employed for melamine detection, while Ag-NiO nanofibrous mat was used for methyl parathion monitoring. Figure 7 shows the corresponding Raman spectra results. It is well-noted that no obvious peak could be observed for both porous Ag and Ag-NiO nanofibrous mat. Figure 7A shows SERS spectra of melamine at various concentrations (0 to 5 × 10^−^^4^ M) recorded on porous Ag. One can see that after loading 2.5 × 10^−^^6^ M of melamine on the SERS substrate, a prominent peak at 684 cm^−1^ was observed, corresponding to the characteristic peak of melamine (ring breathing) [43]. The Raman intensities at 684 cm^−1^ gradually increased with the increasing of melamine concentration. The results demonstrated that the porous Ag network displayed good sensitivity (down to micromolar level) towards melamine detection. SERS spectra of methyl parathion at various concentrations on Ag-NiO nanofibrous mat were collected and are shown in Figure 7B. One peak at 1344 cm^−1^ (bending vibration of C-H) [44] appeared upon addition of 1 × 10^−^^5^ M of methyl parathion. Raman intensities increased significantly with the increase of methyl parathion concentrations. At higher concentrations, a new peak at 1111 cm^−1^ was also observed, corresponding to the stretching vibration of C-N [44]. An acceptable sensitivity of Ag-NiO nanofiber-based SERS substrate was also acquired. These results suggest that as-prepared porous Ag and Ag-NiO nanofibrous mat as SERS substrates display good sensitivities towards target molecules, indicating that the electrospinning-calcination, two-step method offers a new, high performance route in the fabrication of SERS substrates.

## 4. Conclusions

In conclusion, we fabricated two new SERS substrates, porous Ag and Ag-NiO nanofiber, by using a simple, electrospinning-calcination two-step method with AgNO_3_/PVP and AgNO_3_/Ni(NO_3_)_2_/PVP as precursors, respectively. Formation of porous Ag was attributed to partial melting of silver at 500 °C. By contrast, the introduction of Ni(NO_3_)_2_ maintained the nanofibrous structure due to the formation and presence of NiO. The good SERS performances of as-synthesized quasi-three-dimensional porous Ag and Ag-NiO nanofibrous mat were first demonstrated using R6G as a model compound. The feasibility of a porous Ag and Ag-NiO nanofiber-based SERS sensing platform was further demonstrated for monitoring melamine and methyl parathion, respectively, indicating their potential application in food safety and environmental monitoring. These results demonstrate that the electrospinning-calcination two-step method offers a new strategy in the preparation of highperformance SERS substrates.

## Figures and Tables

**Table 1:** Raman scattering peaks assignment for R6G, melamine and methyl parathion

Chemicals	Raman Shift (cm^−1^)	Assignment
Rodamine 6G	769	ip XRD and op C-H bend
	1123	C-H str
	1194	ip XRD, C-H bend, N-H bend
	1311	ip XRB, N-H bend, CH_2_ wag
	1361	XRS, ip C-H bend, C-C str
	1509	XRS, C-N str, C-H bend, N-H bend, C-C str
	1647	XRS, ip C-H bend, C-C str
melamine	684	Ring breathing
methyl parathion	1111	stretching vibration of C-N
	1344	bending vibration of C-H

ip: in plane. op: out of plane. XRD: xanthene ring deformations. XRB: xanthene ring breath. XRS: xanthene ring stretch. str: stretch.

**Table 2:** Values of measured $I_{SERS}$ and $I_{NRS}$ on porous Ag and Ag-NiO nanofibers, as well as $N_{SERS}$ and $N_{NRS}$.

SERS Substrates	$I_{SERS}\;(\mathrm{Counts}\;{\mathrm{mW}}^{-1}\cdot S^{-1})$	$I_{NRS}\;(\mathrm{Counts}\;{\mathrm{mW}}^{-1}\cdot S^{-1})$	$C_{SERS}\;(M)$	$C_{NRS}\;(M)$	EF
Porous Ag	541	68	5 × 10^−8^	1 × 10^−3^	1.59 × 10^5^
Ag-NiO nanofibers	589		3 × 10^−7^		2.89 × 10^4^
